# Role of melatonin in glucose uptake by cardiomyocytes from insulin-resistant Wistar rats

**DOI:** 10.5830/CVJA-2017-018

**Published:** 2017

**Authors:** Nduhirabandi Frederic, Huisamen Barbara, Strijdom Hans, Lochner Amanda, Huisamen Barbara

**Affiliations:** Division of Medical Physiology, Department of Biomedical Sciences, Faculty of Medicine and Health Sciences, Stellenbosch University, South Africa; Division of Medical Physiology, Department of Biomedical Sciences, Faculty of Medicine and Health Sciences, Stellenbosch University, South Africa; Division of Medical Physiology, Department of Biomedical Sciences, Faculty of Medicine and Health Sciences, Stellenbosch University, South Africa; Division of Medical Physiology, Department of Biomedical Sciences, Faculty of Medicine and Health Sciences, Stellenbosch University, South Africa; Biotechnology, Research and Innovation Platform, South African Medical Research Council, Tygerberg, South Africa

**Keywords:** cardiomyocytes, glucose homeostasis, glucose uptake, insulin resistance, melatonin, obesity

## Abstract

**Aim:**

Melatonin supplementation reduces insulin resistance and protects the heart in obese rats. However, its role in myocardial glucose uptake remains unknown. This study investigated the effect of short-term melatonin treatment on glucose uptake by cardiomyocytes isolated from obese and insulin-resistant rats.

**Methods:**

Cardiomyocytes were isolated from obese rats fed a high-calorie diet for 16 to 23 weeks, their age-matched controls, as well as young control rats aged four to eight weeks. After incubation with melatonin with or without insulin, glucose uptake was initiated by the addition of 2-deoxy-D-[3H] glucose and measured after 30 minutes. Additional control and obese rats received melatonin in the drinking water (4 mg/kg/day) for the last six weeks of feeding (20 weeks) and glucose uptake was determined in isolated cardiomyocytes after incubation with insulin. Intraperitoneal glucose tolerance and biometric parameters were also measured.

**Results:**

Obese rats (fed for more than 20 weeks) developed glucose intolerance. Cardiomyocytes isolated from these obese rats had a reduced response to insulin-stimulated glucose uptake (ISGU) (p < 0.05). Melatonin administration *in vitro* had no effect on glucose uptake per se. However, it increased ISGU by cardiomyocytes from the young rats (p < 0.05), while having no effect on ISGU by cardiomyocytes from the older control and obese groups. Melatonin in vivo had no significant effect on glucose tolerance, but it increased basal (p < 0.05) and ISGU by cardiomyocytes from the obese rats (50.1 ± 1.7 vs 32.1 ± 5.1 pmol/mg protein/30 min, p < 0.01).

**Conclusion:**

These data suggest that short-term melatonin treatment *in vivo* but not *in vitro* improved glucose uptake and insulin responsiveness of cardiomyocytes in obesity and insulin-resistance states.

## Aim

Although food shortage and malnutrition are still endemic in low-and middle-income countries,^1^ excessive food intake and reduced physical activity associated with modern lifestyles, as well as night shift-work have led to a dramatic increase in the worldwide prevalence of obesity.^2^,^3^ This is accompanied by various metabolic disorders including, among others, type 2 diabetes and cardiovascular diseases.^4^,^5^ The major basis for this association is the well-known insulin resistance, which is a fundamental aspect in the development of type 2 diabetes and a common pathological link between obesity and cardiac diseases.^6^-^8^ In this condition, the body produces insulin but does not use it properly due to decreased cellular sensitivity to its effect on uptake, metabolism and storage of glucose.^9^

Melatonin or N-acetyl-5-methoxytryptamine is the hormone secreted mainly by the pineal gland during the night. Its role in metabolic diseases has recently attracted many investigators.10 Several animal^11^-^15^ and epidemiological^16^-^20^ studies support the role of melatonin in the regulation of glucose homeostasis. Low melatonin secretion levels are associated with elevated risk for hyperglycaemia and type 2 diabetes.^12^,^18^ Importantly, removal of the melatonin receptor (MT1) significantly impairs the ability of mice to metabolise glucose and induces insulin resistance in these animals,^14^ while melatonin administration improves glucose homeostasis in insulin-resistant animals.^11^,^13^,^21^-^24^ However, the mechanism underlying the role of melatonin in glucose homeostasis is complex and not well understood.^25^

Impairment of insulin-stimulated glucose uptake is considered the most consistent change that develops early in the hearts of animal models of insulin resistance.^26^ This change occurs as a consequence of both reduced glucose transporter 4 (GLUT4) protein expression and impaired translocation.^27^ In this regard, while melatonin’s effects have been extensively reported in other insulin-sensitive organs, such as the hypothalamus, skeletal muscle, liver and adipose tissue,^25^,^28^-^30^ it is unclear whether melatonin affects cardiac glucose uptake in the insulin-resistant state.

A previous study showed that melatonin treatment was able to protect the heart against oxidative damage and restore the expression of the GLUT4 gene as well as glucose uptake of cardiomyocytes isolated from hyperthyroid rats,^31^ supporting the ability of melatonin to improve changes in glucose uptake. Chronic melatonin administration given from the onset of the obesity-inducing diet was recently shown to prevent the harmful effects of obesity, such as insulin resistance and dyslipidaemia and to protect the hearts of obese rats against myocardial ischaemia–reperfusion injury.^32^ In addition, we observed that short-term melatonin consumption also reduced systemic insulin resistance and conferred cardioprotection.^33^ However, whether melatonin treatment affects myocardial insulin sensitivity and glucose uptake remains unknown.

The aim of this study was therefore to investigate the effect of melatonin treatment on myocardial glucose uptake using cardiomyocytes isolated from insulin-resistant rats and their aged-matched controls. To investigate whether melatonin has a direct effect on myocardial glucose uptake, melatonin was administered *in vitro* to isolated cardiomyocytes and in vivo for the measurement of glucose uptake. To evaluate the effect of ageing, cardiomyocytes isolated from normal control rats (seven to eight weeks old) were also included.

## Methods

Sixty male Wistar rats were obtained from the University of Stellenbosch Central Research Facility. They were housed with free access to water and food and a 12-hour dark/light cycle (light from 06:00 to 18:00) with temperature and humidity kept constant at 22oC and 40%, respectively.

The experimental procedure was assessed and approved by the Committee for Ethical Animal Research of the Faculty of Medicine and Health Sciences, University of Stellenbosch (ethical clearance no P08/05/008). Animals were treated according to the *Guide for the Care and Use of Laboratory Animals* published by the US National Institutes of Health (NIH publication No 85–23, revised 1985) and the revised *South African National Standard for the Care and Use of Animals for Scientific Purposes* (South African Bureau of Standards, SANS 10386, 2008).

For evaluation of insulin responsiveness and sensitivity, cardiomyocytes were isolated from (1) normal rats (225−250 g) (n = 12) or (2) diet-induced obese rats (group D) (n = 24) and their age-matched controls (group C) (n = 24) fed a high-calorie diet and standard rat chow, respectively. The high-calorie diet consisted of 65% carbohydrates, 19% protein and 16% fat, while the standard rat chow consisted of 60% carbohydrate, 30% protein and 10% fat.^32^ The diet-induced obese and age-matched control rats were seven to eight weeks old at the onset of the experimental programme, which was continued for a period of 16 to 23 weeks. To evaluate the progressive changes in insulin sensitivity, the feeding regime of our existing model of dietinduced obesity and insulin resistance^32^ was varied from 16 to 23 weeks to exacerbate the effects of obesity, as previously reported.^33^

To determine whether short-term melatonin administration *in vitro* had a direct effect on myocardial glucose uptake, melatonin was administered to the cardiomyocytes after isolation (see below for cardiomyocyte preparation). Briefly, isolated cardiomyocytes were incubated with phloretin (glucose-uptake inhibitor, 400 μM), and melatonin (100 nM) with or without insulin (1–100 nM). Fresh melatonin (Sigma-Aldrich, St Louis, MO, USA) solution was used; melatonin was dissolved in a small quantity of ethanol and then in medium buffer to yield a final concentration of 1 nM, 10 nM, 100 nM, 1 μM or 10 μM (with < 0.005% ethanol). Ethanol at that concentration had no effect on glucose uptake by the cardiomyocytes (results not shown). Phloretin (Sigma-Aldrich, St Louis, MO, USA) was dissolved in dimethyl sulfoxide (DMSO), stored at −80°C as stock, and diluted with medium buffer immediately before use.

To evaluate the effect of in vivo melatonin treatment on myocardial glucose uptake, only rats fed for 20 weeks were used. While studying the effect of *in vitro* melatonin treatment, we observed that compared to their age-matched control rats, only cardiomyocytes isolated from obese rats fed for more than 20 weeks showed a significant decrease in insulin-stimulated glucose uptake (Fig. 3). Four groups were studied including: (1) untreated control (C), (2) treated control (CM), (3) untreated diet (D), and (4) treated diet (DM).

Melatonin was orally administered in the drinking water (4 mg/ kg/day) for six weeks starting from the 14th week of feeding, as described previously.^32^,^33^ This is the lowest concentration to have a significant effect in our model of diet-induced obesity.^33^ Drinking water with or without melatonin was replaced every day one hour before lights off (18:00) and was available throughout the light and dark cycles.^33^ In contrast to humans, rats are active during the night, when their blood melatonin levels are high. A period of six weeks has been shown as the shortest to elicit marked effects of melatonin on the hearts from diet-induced obese rats and to reverse several of the harmful effects of obesity.^33^

Animals were anaesthetised with sodium pentobarbitone (160 mg/kg, intraperitoneally). The hearts were immediately removed and perfused for isolation of cardiomyocytes, as described previously.^34^ The body weight and visceral fat mass were recorded. Adiposity index was calculated as the ratio of visceral fat mass to body weight, multiplied by 100.^3^

Blood glucose levels were determined in the fasting state, as described previously,^35^ at the same time (10:00–12:00). Blood was obtained via a tail prick and levels were determined using a conventional glucometer (Cipla MedPro, Bellville, South Africa). Intraperitoneal glucose tolerance (IPGT) curves were generated in animals after an overnight fasting period. Animals were injected with 1 g/kg of a 50% sucrose solution and blood glucose levels were recorded over a two-hour period.

Calcium-tolerant adult ventricular myocytes were isolated from the different animal groups, as previously reported.^34^ After isolation, the myocytes were suspended in a medium buffer containing (in mM): HEPES 10, KCL 6, NaH2PO4 0.2, Na2HPO4 1, MgSO4 1.4, NaCl 128, pyruvate 2, glucose 5.5, and 2% BSA (fraction V, fatty acid free) plus calcium 1.25 mM, at pH 7.4. The cells were left for one to two hours under an oxygen atmosphere on a gently shaking platform to recover from the trauma of isolation. After recovery, the cells were allowed to settle into a loose pellet and the supernatant was removed. This procedure routinely rendered in excess of 80% viable cells, as measured by trypan blue exclusion. They were additionally washed twice with and suspended in a suitable volume of the above medium buffer but without glucose and pyruvate for subsequent glucose uptake determinations.

Cardiomyocyte glucose uptake was measured essentially as described previously^34^ in a final assay volume of 750 μl. Cells prepared from the different groups of animals were incubated with or without one, 10 or 100 nM insulin for 30 minutes. After a total incubation period of 45 minutes, glucose uptake was initiated by addition of 2-deoxy-D-[3H] glucose (2DG) (1.5 μCi/ ml; final concentration 1.8 μM) (Perkin Elmer, Boston, USA). Glucose uptake was allowed to progress for 30 minutes before stopping the reaction by adding phloretin (final concentration 400 μM). Thereafter, the cells were centrifuged at 1 000 g for one minute and the supernatant containing radiolabelled 2DG was aspirated. The subsequent pellet was washed twice with medium buffer without substrate and then dissolved in 0.5 M NaOH; 50 μl of this solution was used for the determination of the protein content by the method of Lowry et al.,^36^ while the rest was counted for radioactivity using a scintillation counter (Beckman).

The Western blot technique was performed as previously reported, using the whole heart tissue33 and isolated cardiomyocytes.^34^ Cell lysates were made after 30 minutes’ incubation with or without insulin or melatonin (before the addition of 2DG). Thereafter the cells were put on ice, transferred to Eppendorf tubes, quickly centrifuged and washed three times with ice-cold medium buffer without substrate. The resultant cell pellet was then lysed in 100 μl of lysis buffer.^34^ At this point the cells were sonicated on ice (three times, intervals of three-second pulses with one-second break) and centrifuged for 20 minutes. The subsequent pellet was discarded and the supernatant used as cell lysate for Western blotting.

Total and phospho PKB/Akt (Ser-473) expressions were evaluated in the cardiomyocytes after incubation with melatonin with or without insulin, as previously described.^34^ In addition, GLUT4 expression was evaluated in whole heart lysates after six weeks of melatonin treatment, as previously described.^33^ All antibodies were purchased from Cell Signaling (USA). Betatubulin was used as a loading control. Protein activation is expressed in arbitrary densitometry units as phospho/total ratios.

## Statistical analysis

Data are expressed as mean ± standard error of the mean (SEM). When comparisons between two groups (treated and untreated) were made, an unpaired Student’s t-test was performed. For multiple comparisons, the ANOVA (two-way when appropriate), followed by the Bonferroni correction was applied. Statistical significance was considered for a p-value < 0.05.

## Results

## Effect of melatonin treatment *in vitro* on glucose uptake by cardiomyocytes

**Fig. 1:**
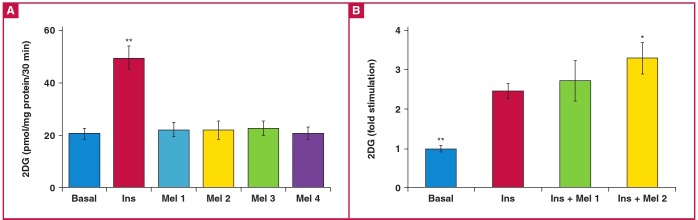
Effect of *in vitro* melatonin treatment on basal and insulin-stimulated glucose uptake by cardiomyocytes from young control rats (dose response). Cardiomyocytes were isolated and incubated with melatonin and/or insulin for a period of 30 minutes. The accumulated radiolabelled 2 deoxyglucose (2DG) was measured using a scintillation counter and expressed as pmol/ mg protein/30 min. A: Effect on basal glucose uptake. Ins: insulin (1 nM), Mel: melatonin (Mel 1: 10 nM, Mel 2: 100 nM, Mel 3: 10 μM, Mel4: 50 μM), ^**^p < 0.01 (vs basal or melatonin), n (individual preparations): n = 12 (basal), 11 (Ins), three (Mel 1), eight (Mel 2), four (Mel 3), three (Mel 4); analysed in duplicate. B: Effect on insulin-stimulated glucose uptake (fold stimulation). Ins: insulin (1 nM), Mel: melatonin (Mel 1: 10 nM, Mel2: 100 nM); ^*^p < 0.05 (Ins vs Ins + Mel 2); ^**^p < 0.05 (basal vs Ins or Ins + Mel 1 or 2); n = 12 (basal), 11 (Ins), five (Ins + Mel 1), six (Ins + Mel 2) individual preparations/group; analysed in duplicate.

Compared to basal levels, melatonin treatment (10 and 100 nM, 10 and 50 μM) had no significant effect on glucose uptake by the cardiomyocytes isolated from normal rats (Fig. 1A). Insulin (1 nM) administration alone caused a 2.3-fold increase in glucose uptake compared to basal levels (Fig. 1B). However, when insulin was added to cells treated with melatonin (100 nM), there was a further stimulation of glucose uptake (3.4 ± 0.5- vs 2.5 ± 0.2-fold increase, p < 0.05) (Fig. 1B). As melatonin at other concentrations (10 nM) did not influence the levels of insulinstimulated glucose uptake (Fig. 1B) when compared to insulin alone, only 100 nM was used in subsequent experiments.

**Fig. 2:**
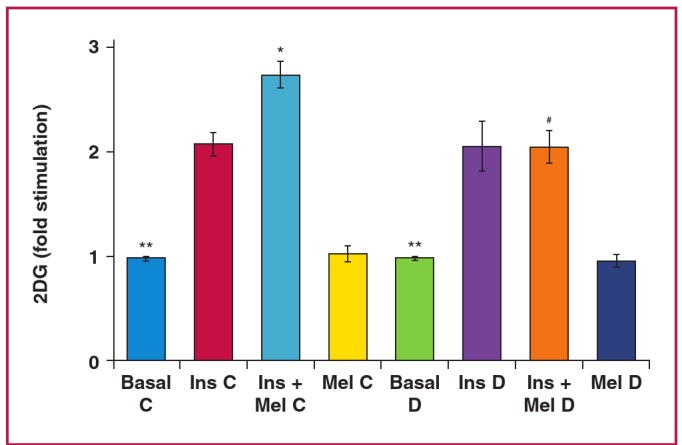
Effect of *in vitro* melatonin treatment on insulin-stimulated glucose uptake of cardiomyocytes isolated from control (C) and high-calorie diet (diet-induced obesity) (D) groups after 16 to 19 weeks. 2DG: 2 deoxyglucose, Ins: insulin (1 nM), Mel: melatonin (100 nM); ^*^p < 0.05 (Ins C vs Ins + Mel C), ^**^p < 0.01(basal vs Ins or Ins + Mel; Ins C vs Ins D), ^#^p < 0.05 (Ins + Mel D vs Ins + Mel C), n = four to six individual preparations/group; analysed in duplicate.

Cardiomyocytes isolated from the control (C) and obese (D) rats after 16 to 19 weeks of feeding, exhibited no significant difference in basal as well as insulin-stimulated glucose uptake between the two groups (Table 1,Fig. 2).As was observed in cardiomyocytes isolated from normal rats (Fig. 1A), melatonin administration (100 nM) also had no significant effect on basal glucose uptake in group C and D rats fed for 16 to 19 weeks (Table 1). However, it enhanced the insulin-stimulated glucose uptake in group C compared to group D rats (C: 73.9 ± 4.1 vs D: 47.5 ±; 4.9 pmol/mg protein/30 min, p < 0.05) (Table 1, Fig. 2).

**Table 1:** Body weight and visceral mass of rats fed for 16 to 19 weeks and their corresponding glucose uptake by the cardiomyocytes

	*Body weight and visceral fat mass*			*Glucose uptake (pmol/mg protein/30 min)*			
*Group*	*Body weight (g)*	*Visceral fat (g)*	*Adiposity index*	*Basal*	*nsulin*	*Ins + Mel*	*Mel*
C	435 ± 21	17.0 ± 1.4	3.8 ± 0.18	25.6 ± 2.8	49.3 ± 5.6^*^	73.9 ± 4.1^***#^	25.5 ± 4.4
D	517 ± 11^###^	33.3 ± 1.3^###^	6.39 ± 0.3^###^	20.8 ± 3.1	40.8 ± 3.8^*^	47.5 ± 4.9^*^	20.0 ± 3.4
n	6	6	6	6	6	4	6

C: control, D: high-calorie diet, adiposity index = [(visceral fat/body weight) × 100], Ins: insulin (1 nM), Mel: melatonin (100 nM), ^*^p < 0.05 (vs basal), ^***^p < 0.001 (vs basal), ^#^p < 0.05 (vs D), ^###^p < 0.001 (vs C), n = four to six individual preparations per group, uptake determined in duplicate for each preparation.

**Fig. 3:**
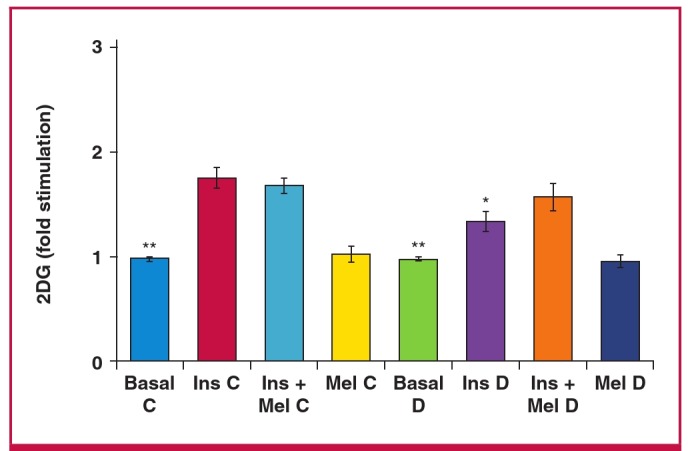
Effect of *in vitro* melatonin treatment on insulin-stimulated glucose uptake of cardiomyocytes isolated from control (C) and high-calorie diet (diet-induced obesity) (D) groups after 20 to 23 weeks. 2DG: 2 deoxyglucose, Ins: insulin (1 nM), Mel: melatonin (100 nM); ^*^p < 0.05 (Ins C vs Ins D), ^**^p < 0.01 (basal vs Ins or Ins + Mel), n = four to six individual preparations/group; analysed in duplicate.

After 20 to 23 weeks of feeding, although the diet had no significant effect on basal glucose uptake by isolated cardiomyocytes (Table 2), insulin-stimulated glucose uptake was significantly lower in group D rats compared with the control group (C: 35.3 ± 6.3 vs D: 25.9 ± 1.6 pmol/mg protein/30 min, p < 0.05) (Fig. 3), while melatonin treatment had no effect on insulinstimulated glucose uptake in both group C and D rats (Fig. 3).

## Effect of melatonin treatment in vivo on glucose uptake by insulin-resistant cardiomyocytes

After 20 to 23 weeks, rats fed a high-calorie diet exhibited significantly increased body weight (C: 433 ± 25 vs D: 538 ± 43 g, p < 0.05), visceral fat mass (C: 17.7 ± 1.8 vs D: 37.5 ± 7.5 g, p < 0.001) as well as adiposity index (Table 3). Melatonin treatment for six weeks reduced body weight and adiposity index values in group D rats (p < 0.05) (Table 3).

**Table 3:** Body weight, visceral fat mass and IPGT

*Parameters*	*C*	*CM*	*D*	*DM*
Body weight (g)	433 ± 25	411 ± 17	538 ± 43^***^	488 ± 21^#^
Visceral fat (g)	17.7 ± 1.8	14.33 ± 1.9^*^	37.50 ± 7.5^***^	28 ± 4^#^
Adiposity index	4.1 ± 0.2	3.4 ± 0.16^*^	6.9 ± 0.23^***^	5.7 ± 0.3^#^
AUC for IPGT	761.5 ± 27.7	760.2 ± 38.8	870.7 ± 25.2^*^	826.7 ± 32.5
n	6	6	6	6

C: control, D: high-calorie diet, CM and DM: control and diet receiving melatonin for six weeks, adiposity index [(visceral fat/body weight) × 100], AUC: area under the curve, IPGT: intraperitoneal glucose tolerance, *p < 0.05 (vs C), ***p < 0.001(vs C), #p < 0.05 (vs D), n = six per group.

**Fig. 4:**
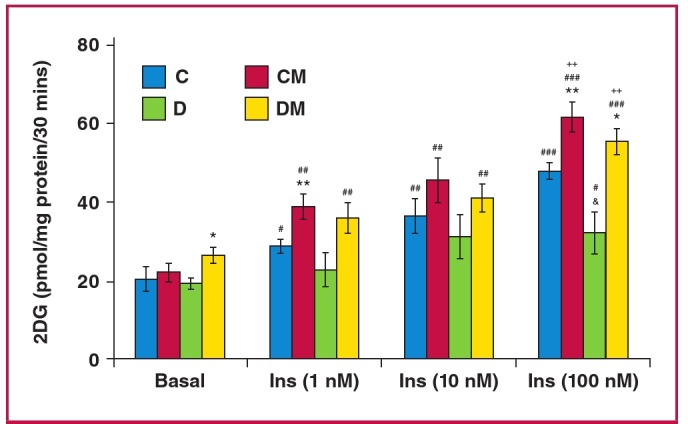
Effect of in vivo melatonin treatment (for the last six weeks of feeding) on insulin-stimulated glucose uptake by cardiomyocytes isolated from rats fed a high-calorie diet (20 weeks). Cardiomyocytes were isolated and stimulated with increasing concentrations of insulin for a period of 30 minutes. The accumulated radiolabelled 2DG was measured and expressed as pmol/mg protein/ 30 min. Ins: insulin, C: control, CM: control with melatonin, D: high-calorie diet (dietinduced obesity), DM: diet with melatonin. Treated vs untreated (same dose of insulin or basal): *p < 0.05 (DM vs D), **p < 0.01 (CM vs C). Different doses of insulin vs basal (same group of treatment): #p < 0.05 vs basal, ##p < 0.01 vs basal, ###p < 0.001 vs basal. C vs D (same dose of insulin): and p < 0.05 (D vs C). Comparison between different doses of insulin (same group of treatment): ++p < 0.01 vs 1 nM Ins, n = four to six individual preparations/group; analysed in duplicate.

To evaluate the glucose uptake by cardiomyocytes from control and obese rats, a dose response with increasing concentrations of insulin was performed (Fig. 4). The diet had no effect on basal glucose uptake by cardiomyocytes isolated from both group C and D rats (Fig. 4). However it reduced insulin-stimulated glucose uptake in group D rats (Fig. 4, Table 2). Oral melatonin treatment in vivo for six weeks increased the basal glucose uptake by cardiomyocytes from group D rats (DM: 26.4 ± 2.1 vs D: 19.8 ± 3.4 pmol/mg protein/30 min, p < 0.05) while having no effect in group C rats (CM: 22.6 ± 3.7 vs C: 21.1 ± 3.5 pmol/mg protein/30min, p > 0.05) (Fig. 4). Additionally, compared to their respectiveuntreated group, cardiomyocytes isolated from the controltreatment group (CM) had elevated insulin-stimulated glucoseuptake (p < 0.05) (Fig. 4). Furthermore, cardiomyocytes fromthe D treatment group (DM) also showed a further elevation ofinsulin-stimulated glucose uptake with insulin administration (100 nM), compared to the untreated group (DM: 50.1 ± 1.7 vs D: 32.1 ± 5.1 pmol/mg protein/30 min, p < 0.01) (Fig. 4).

**Table 2:** Body weight and visceral mass of rats fed for 20 to 23 weeks and their corresponding glucose uptake by the cardiomyocytes

	*Body weight and visceral fat mass*			*Glucose uptake (pmol/mg protein/30 min)*			
*Group*	*Body weight (g)*	*Visceral fat (g)*	*Adiposity index*	*Basal*	*Insulin*	*Ins + Mel*	*Mel*
C	457 ± 14	18.4 ± 10.9	4 ± 0.2	19.9 ± 2.6^**^	35.3 ± 6.3^#^	33.5 ± 5.9>	*19.2 ± 1.7*
D	575 ± 61^###^	38.7 ± 2.6^###^	6.7 ± 0.6^###^	18.1 ± 1.6^**^	25.9 ± 1.6	27.8 ± 1.1	18.4 ± 2.3
n	6	6	6	6	6	5	6

C: control, D: high-calorie diet, adiposity index [(visceral fat/body weight) × 100], Ins: insulin (1 nM), Mel: melatonin (100 nM), ^**^p < 0.01 (vs Ins or Ins + Mel), ^#^p < 0.05 (vs D), ^###^p < 0.001 (vs C), n = five to six individual preparations per group, uptake determined in duplicate for each preparation.

## Effect of melatonin treatment in vivo on IPGT test in insulin-resistant rats

**Fig. 5:**
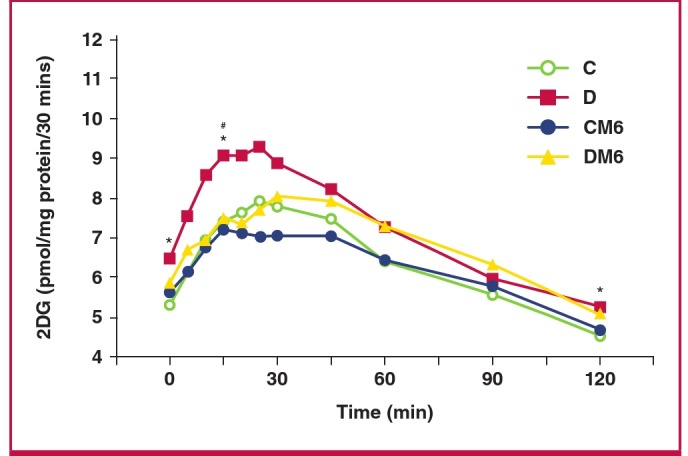
Effect of in vivo melatonin treatment (for the last six weeks of feeding) on intraperitoneal glucose tolerance. C: control, CM6: control with six weeks’ melatonin treatment, D: high-calorie diet (diet-induced obesity), DM6: high-calorie diet with six weeks’ melatonin treatment, *p < 0.05 (D vs C), #p < 0.05 (D vs DM6), n = six per group.

A high-calorie diet increased basal fasting blood glucose levels compared to the control diet (5.2 ± 0.28 vs 6.4 ± 0.17 mM, p < 0.05). Similarly, at the end of the test, group D rats continued to have elevated glucose levels (4.5 ± 0.2 vs 5.2 ± 0.1 mM, p < 0.05), compared to the control group (Fig. 5). The area under the curve was also elevated in group D rats, compared to the controls (870.7 ± 25.6 vs 761.8 ± 27.7, p < 0.05) (Table 3). However, despite a significant decrease in blood glucose levels in the melatonin-treated D rats observed between 15 and 25 minutes of the test, we noted that melatonin treatment had no significant effect on basal glucose levels and the overall area under curve in both groups (Fig. 5).

## Discussion

Our aim was to investigate the effect of melatonin treatment on basal glucose uptake and insulin responsiveness as indicated by glucose uptake, using cardiomyocytes isolated from young control rats, age-matched controls and obese, insulin-resistant rats. The results indicated that (1) melatonin treatment *in vitro* had no effect on glucose uptake but increased insulin-stimulated glucose uptake by cardiomyocytes from only the young and age-matched control rats (Fig. 1B, Table 1); (2) melatonin treatment in vivo increased basal and insulin-stimulated glucose uptake by cardiomyocytes isolated from the hearts of obese, insulin-resistant rats.

During the basal state, glucose transport is commonly considered the rate-limiting step for muscle glucose metabolism.^37^ The involvement of melatonin in glucose uptake was supported by the observation that pinealectomised animals develop insulin resistance associated with a decrease in glucose uptake by adipose tissue.^15^,^38^ Accordingly, administration of melatonin reversed pinealectomy-induced insulin resistance and improved glucose uptake by isolated adipose tissue.^15^,^38^ In contrast to this, our data show that melatonin per se had no significant effect on *in vitro* glucose uptake by cardiomyocytes isolated from young normal or obese rats and their age-matched controls (Fig. 1A, Tables 1, 2). A similar observation was previously reported in rat skeletal muscle cells39 and chick brain,40 as well as in adipose tissue from a female fruit bat.^41^

Of interest was our finding that acute melatonin administration *in vitro* enhanced insulin-stimulated glucose uptake by cardiomyocytes from normal young rats (Fig 1B) as well as the control rats fed for 16 to 19 weeks (Fig. 2). The enhanced insulin responsiveness of glucose uptake may be related to a synergistic interaction between melatonin and insulin action, supporting the insulin-sensitising effect by melatonin, as previously demonstrated.^39^,^41^,^42^

The *in vitro* melatonin-enhancing effect on insulin-stimulated glucose uptake was not observed in cardiomyocytes isolated from either the control or obese groups fed for more than 20 weeks (Fig. 3), indicating a progressive loss of the synergistic interaction between melatonin and insulin action. Although this is difficult to explain, it may have resulted from ageing in the control group, as previously demonstrated.^43^ On the other hand, cardiomyocytes from obese animals fed for 16 to 19 weeks were almost as insulinresponsive as the control cardiac myocytes, but did not exhibit the potentiating effect of melatonin compared to the control group.

Various physiological factors such as an effect on adiponectin and leptin may have contributed to the overall effect of in vivo melatonin on glucose uptake, as previously discussed.^10^ In a preventative-treatment setting, 16 weeks of melatonin consumption, starting before the establishment of obesity, reduced hypertriglyceridaemia and increased high-density lipoprotein cholesterol levels in rats fed the same high-calorie diet.^32^ However, the exact mechanism whereby in vivo melatonin treatment affects glucose homeostasis and enhances insulin responsiveness is complex and not fully elucidated.

Melatonin induced a significant reduction in body weight, associated with a concomitant increase in basal glucose uptake by isolated cardiomyocytes from the obese rats. This effect is consistent with previous observations that chronic melatonin treatment reduced body weight gain and insulin resistance in mice^11^ and rats^21^ fed a high-fat diet, as well as in old obese^28^ and young Zucker diabetic fatty^13^ rats. Therefore, melatonin action may involve melatonin receptors and various indirect effects on the liver, pancreas and other peripheral insulin-sensitive organs, such as adipose tissue and skeletal muscle.^25^ A recent report shows that the removal of melatonin receptors (MT1 or MT2) in mice abolished the daily rhythm in blood glucose levels,^44^ confirming the role of melatonin signalling in the control of glucose homeostasis.

Contrary to the *in vitro* situation, melatonin administered in vivo increased basal glucose uptake by cardiomyocytes isolated from obese rats. Mechanistically, this may involve glucose transporter 1 (GLUT1), which is usually associated with basal glucose uptake by cardiomyocytes, and its expression would give more insight.^45^ Therefore, it may be that there was an increase in the expression or membrane translocation of GLUT1 in these cardiomyocytes from obese rats treated with melatonin. In addition, insulin was able to elicit a significant response in untreated control animals, while this was not the case in the obese animals after 20 to 23 weeks. This observation could be explained by the insulin-resistant state of the cells from the obese animals compared to their controls. Interestingly, cardiomyocytes prepared from control as well as obese animals treated with melatonin showed a significantly higher response to insulin than the untreated counterparts (Fig. 4).

With regard to the effect of melatonin on glucose tolerance, the present data show that obese rats developed glucose intolerance, and melatonin had no effect on basal glucose levels (10:00–12:00). While data on nocturnal glucose levels may be different, six-week melatonin treatment also reduced systemic insulin resistance in obese rats without affecting basal fasting blood glucose levels.33 These results are consistent with previous findings:46 between 15 and 25 minutes following glucose injection, obese melatonin-treated rats had a significant decrease in blood glucose levels compared to the untreated obese group, somehow indicating their increased ability to absorb glucose.

The reduction in insulin resistance or improved glucose uptake and utilisation may involve changes in the metabolic profile, such as increasing adiponectin levels after long-13,23 and short-term^33^ melatonin administration. Melatonin-induced beneficial changes in adipose tissue^41^,^47^ may in turn additionally contribute to improved whole-body insulin sensitivity. Moreover, as indicated above, melatonin may improve glucose homeostasis via its actions in the hypothalamus and liver.^48^

Impairment of insulin-stimulated glucose transport is considered the most consistent change that develops early in the hearts of animal models of insulin resistance.26 Since GLUT4 is the most prominent glucose transporter in differentiated cardiomyocytes,^49^ our data underscore the importance of further investigation analysing the expression of intermediates of insulin signal transduction and the effects of melatonin treatment thereupon in cardiomyocytes isolated from treated control and obese hearts.

The effect of six weeks of melatonin treatment on the basal expression and activation of a number of intermediates in myocardial tissue from control and obese rats has been studied previously in our laboratory: baseline activation of PKB/ Akt, extracellular signal-regulated kinase (ERK) p42/p44 and glycogen synthase kinase 3 beta (GSK3β) were found to be significantly upregulated by melatonin treatment in both control and obese rats.33 However, it will be also important to determinewhether these observed beneficial changes were secondary to theimproved whole-body insulin sensitivity or whether there werechanges in cardiomyocyte protein expression and activation perse elicited by melatonin treatment.

**Fig. 6:**
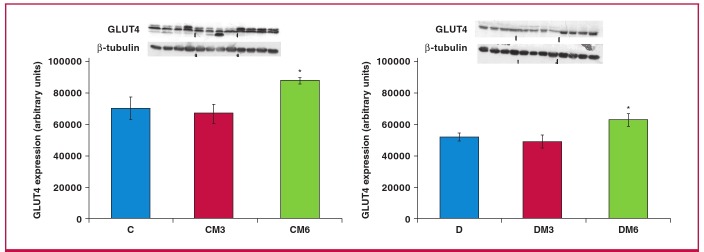
The effects of melatonin treatment on GLUT4 expression after three and six weeks of treatment. Hearts were isolated fromrats fed a high-calorie diet for 20 weeks and their age-matched controls. Both control and obese groups received drinkingwater with/without melatonin (4 mg/kg/day) for three or six weeks starting after 14 weeks of feeding. C: control group, D: highcaloriediet (obesity) group; CM3, DM3, CM6 and DM6: group C and D rats receiving melatonin treatment for three weeks(M3) or six weeks (M6); beta-tubulin was used as a loading control. C and D performed on the different blot (p > 0.05 C vsD), *p < 0.05 (CM6 vs C) or DM6 vs D, n = four hearts/group.

**Fig. 7:**
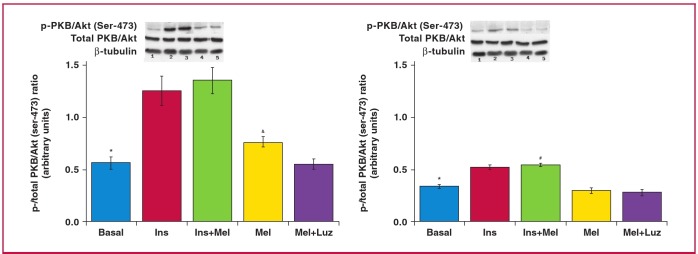
Effects of *in vitro* melatonin administration to isolated cardiomyocytes on PKB/Akt expression and phosphorylation (rats fed for 20 weeks). Cardiomyocytes were isolated and incubated with melatonin with or without insulin stimulation. C: control, D: high-calorie diet. 1: basal, 2: Ins (insulin), 3: Insulin + melatonin, 4: Mel (melatonin), 5: luzindole + melatonin, Luz (luzindole), C: *p < 0.05 (Ins or Ins + Mel vs basal), and p < 0.05 (Mel vs basal or Mel + Luz), D: *p < 0.05 (Ins or Ins + Mel vs basal), #p < 0.05 (D vs C), n = three individual preparations/group. Blots are representative. Beta-tubulin was used as a loading control. C and D performed on the same blot.

In this regard, a marginal increase in GLUT4 expression was previously reported to be associated with an increase in glucose uptake by melatonin-treated adipose tissue.^41^ Our additional observations showed significant increases in GLUT4 expression in the whole heart tissue of obese rats after six weeks of in vivo melatonin treatment (Fig. 6). Interestingly, as expected, the significant lowering in glucose uptake by cardiomyocytes from obese rats was also reflected in the reduction in PKB/Akt activation when compared with their age-matched controls (Fig. 7).

## Conclusion

To our knowledge, this is the first study on the role of melatonin in cardiac glucose uptake in an insulin-resistant state. The cardiovascular benefits of melatonin supplementation are supported by the fact that circulating melatonin levels are decreased in cardiovascular diseases.50,51 Convincing evidence exists for the benefits of increasing glucose uptake as an important therapeutic goal in the management of left ventricular systolic dysfunction.52 Although its role in melatonin-induced cardioprotection needs further investigation, present data suggest that short-term melatonin treatment in vivo, but not *in vitro*, improved basal glucose uptake and insulin responsiveness in insulin-resistant cardiomyocytes isolated from obese rats.

## References

[1] Muzigaba M, Puoane T, Sanders D (2016). The paradox of undernutrition and obesity in South africa: A contextual overview of food quality, access and availability in the new democracy. In: Caraher M, Coveney J, eds. Food Poverty and Insecurity: International Food Inequalities.

[2] Finucane MM, Stevens GA, Cowan MJ (2011). National, regional, and global trends in body-mass index since 1980: Systematic analysis of health examination surveys and epidemiological studies with 960 country-years and 9·1 million participants.. Lancet.

[3] Rybnikova NA, Haim A, Portnov BA (2016). Does artificial light-at-night exposure contribute to the worldwide obesity pandemic?. Int J Obes (Lond).

[4] Guh DP, Zhang W, Bansback N, Amarsi Z, Birmingham CL, Anis AH (2009). The incidence of co-morbidities related to obesity and overweight: A systematic review and meta-analysis.. BMC Public Health.

[5] Scheen AJ, Van Gaal LF (2014). Combating the dual burden: Therapeutic targeting of common pathways in obesity and type 2 diabetes. Lancet Diabetes Endocrinol.

[6] Reaven GM (2011). Insulin resistance: The link between obesity and cardiovascular disease. Med Clin North Am.

[7] Benito M (2011). Tissue specificity on insulin action and resistance: Past to recent mechanisms. Acta Physiol (Oxf).

[8] Riehle C, Abel ED (2016). Insulin signaling and heart failure. Circ Res.

[9] Hardy OT, Czech MP, Corvera S (2012). What causes the insulin resistance underlying obesity?. Curr Opin Endocrinol Diabetes Obes.

[10] Nduhirabandi F, du Toit EF, Lochner A (2012). Melatonin and the metabolic syndrome: A tool for effective therapy in obesity-associated abnormalities?. Acta Physiol (Oxf).

[11] Sartori C, Dessen P, Mathieu C (2009). Melatonin improves glucose homeostasis and endothelial vascular function in high-fat diet-fed insulin-resistant mice. Endocrinology.

[12] Peschke E, Frese T, Chankiewitz E (2006). Diabetic goto kakizaki rats as well as type 2 diabetic patients show a decreased diurnal serum melatonin level and an increased pancreatic melatonin-receptor status. J Pineal Res.

[13] Agil A, Rosado I, Ruiz R, Figueroa A, Zen N, Fernandez-Vazquez G (2012). Melatonin improves glucose homeostasis in young zucker diabetic fatty rats. J Pineal Res.

[14] Contreras-Alcantara S, Baba K, Tosini G (2010). Removal of melatonin receptor type 1 induces insulin resistance in the mouse. Obesity (Silver Spring).

[15] Lima FB, Machado UF, Bartol I (1998). Pinealectomy causes glucose intolerance and decreases adipose cell responsiveness to insulin in rats. Am J Physiol Endocrinol Metab.

[16] Xia Q, Chen ZX, Wang YC (2012). Association between the melatonin receptor 1B gene polymorphism on the risk of type 2 diabetes, impaired glucose regulation: A meta-analysis. PLoS One.

[17] Ronn T, Wen J, Yang Z (2009). A common variant in MTNR1B, encoding melatonin receptor 1B, is associated with type 2 diabetes and fasting plasma glucose in han chinese individuals. Diabetologia.

[18] McMullan CJ, Schernhammer ES, Rimm EB, Hu FB, Forman JP (2013). Melatonin secretion and the incidence of type 2 diabetes. J Am Med Assoc.

[19] McMullan CJ, Curhan GC, Schernhammer ES, Forman JP (2013). Association of nocturnal melatonin secretion with insulin resistance in nondiabetic young women. Am J Epidemiol.

[20] Prokopenko I, Langenberg C, Florez JC (2009). Variants in MTNR1B influence fasting glucose levels. Nat Genet.

[21] Wan X, Li S, Xi S, Wang v, Guo Y, Wang X (2013). Long-term melatonin administration improves glucose homeostasis and insulin resistance state in high-fat-diet fed rats. Cent Eur J Biol.

[22] Shieh JM, Wu HT, Cheng KC, Cheng JT (2009). Melatonin ameliorates high fat diet-induced diabetes and stimulates glycogen synthesis via a PKCzetaakt-GSK3beta pathway in hepatic cells. J Pineal Res.

[23] Kitagawa A, Ohta Y, Ohashi K (2012). Melatonin improves metabolic syndrome induced by high fructose intake in rats. J Pineal Res.

[24] She M, Deng X, Guo Z (2009). NEU-P11, a novel melatonin agonist, inhibits weight gain and improves insulin sensitivity in high-fat/highsucrose-fed rats. Pharmacol Res.

[25] Karamitri A, Renault N, Clement N, Guillaume J, Jockers R (2013). Minireview: toward the establishment of a link between melatonin and glucose homeostasis: Association of melatonin MT2 receptor variants with type 2 diabetes. Molec Endocrinol.

[26] Wright JJ, Kim J, Buchanan J (2009). Mechanisms for increased myocardial fatty acid utilization following short-term high-fat feeding.. Cardiovasc Res.

[27] Cook SA, Varela-Carver A, Mongillo M (2010). Abnormal myocardial insulin signalling in type 2 diabetes and left-ventricular dysfunction. Eur Heart J.

[28] Zanuto R, Siqueira-Filho MA, Caperuto LC (2013). Melatonin improves insulin sensitivity independently of weight loss in old obese rats. J Pineal Res.

[29] Anhe GF, Caperuto LC, Pereira-Da-Silva M (2004). In vivo activation of insulin receptor tyrosine kinase by melatonin in the rat hypothalamus. J Neurochem.

[30] Agil A, El-Hammadi M, Jimenez-Aranda A (2015). Melatonin reduces hepatic mitochondrial dysfunction in diabetic obese rats. J Pineal Res.

[31] Ghosh G, De K, Maity S (2007). Melatonin protects against oxidative damage and restores expression of GLUT4 gene in the hyperthyroid rat heart. J Pineal Res.

[32] Nduhirabandi F, du Toit EF, Marais D, Lochner A (2011). Chronic melatonin consumption prevents obesity-related metabolic abnormalities and protects the heart against myocardial ischemia and reperfusion injury in a prediabetic model of diet-induced obesity. J Pineal Res.

[33] Nduhirabandi F, Huisamen B, Strijdom H, Blackhurst D, Lochner A (2014). Short-term melatonin consumption protects the heart of obese rats independent of body weight change and visceral adiposity. J Pineal Res.

[34] Huisamen B, Donthi RV, Lochner A (2001). Insulin in combination with vanadate stimulates glucose transport in isolated cardiomyocytes from obese zucker rats. Cardiovasc Drugs Ther.

[35] Huisamen B, Dietrich D, Bezuidenhout N (2012). Early cardiovascular changes occurring in diet-induced, obese insulin-resistant ratios. Mol Cell Biochem.

[36] Lowry OH, Rosebrough NJ, Farr AL, Randall RJ (1951). Protein measurement with the folin phenol reagent. J Biol Chem.

[37] Fueger PT, Shearer J, Bracy DP (2005). Control of muscle glucose uptake: Test of the rate-limiting step paradigm in conscious, unrestrained mice. J Physiol.

[38] Alonso-Vale MI, Anhe GF, Borges-Silva CN (2004). Pinealectomy alters adipose tissue adaptability to fasting in rats. Metabolism.

[39] Teodoro BG, Baraldi FG, Sampaio IH (2014). Melatonin prevents mitochondrial dysfunction and insulin resistance in rat skeletal muscle. J Pineal Res.

[40] Cantwell EL, Cassone VM (2002). Daily and circadian fluctuation in 2-deox [14C]-glucose uptake in circadian and visual system structures of the chick brain: Effects of exogenous melatonin. Brain Res Bull.

[41] Banerjee A, Udin S, Krishna A (2011). Regulation of leptin synthesis in white adipose tissue of the female fruit bat, cynopterus sphinx: role of melatonin with or without insulin. Exp Physiol.

[42] Wang P, She M, He P (2013). Piromelatine decreases triglyceride accumulation in insulin resistant 3T3-L1 adipocytes: Role of ATGL and HSL. Biochimie.

[43] Carroll R, Carley AN, Dyck JR, Severson DL (2005). Metabolic effects of insulin on cardiomyocytes from control and diabetic db/db mouse hearts. Am J Physiol Endocrinol Metab.

[44] Owino S, Contreras-Alcantara S, Baba K, Tosini G (2016). Melatonin signaling controls the daily rhythm in blood glucose levels independent of peripheral clocks. PloS One.

[45] Mueckler M, Thorens B (2013). The SLC2 (GLUT) family of membrane transporters. Mol Aspects Med.

[46] Rios-Lugo MJ, Cano P, Jimenez-Ortega V (2010). Melatonin effect on plasma adiponectin, leptin, insulin, glucose, triglycerides and cholesterol in normal and high fat-fed rats. J Pineal Res.

[47] Zanquetta MM, Seraphim PM, Sumida DH, Cipolla-Neto J, Machado UF (2003). Calorie restriction reduces pinealectomy-induced insulin resistance by improving GLUT4 gene expression and its translocation to the plasma membrane. J Pineal Res.

[48] Faria JA, Kinote A, Ignacio-Souza LM (2013). Melatonin acts through MT1/MT2 receptors to activate hypothalamic AKT and suppress hepatic gluconeogenesis in rats. Am J Physiol Endocrinol Metab.

[49] Montessuit C, Lerch R (2013). Regulation and dysregulation of glucose transport in cardiomyocytes. Biochim Biophys Acta.

[50] Dominguez-Rodriguez A, Abreu-Gonzalez P, Arroyo-Ucar E, Reiter RJ (2012). Decreased level of melatonin in serum predicts left ventricular remodelling after acute myocardial infarction. J Pineal Res.

[51] Dominguez-Rodriguez A, Abreu-Gonzalez P, Garcia MJ, Sanchez J, Marrero F, de Armas-Trujillo D (2002). Decreased nocturnal melatonin levels during acute myocardial infarction. J Pineal Res.

[52] Fields AV, Patterson B, Karnik AA, Shannon RP (2009). Glucagon-like peptide-1 and myocardial protection: More than glycemic control. Clin Cardiol.

